# Apoptotic potential of C-phycoerythrin from Phormidium sp. A27DM and Halomicronema sp. A32DM on human lung carcinoma cells

**DOI:** 10.17179/excli2014-696

**Published:** 2015-04-10

**Authors:** Datta Madamwar, Dipak K Patel, Swati N Desai, Kapil K Upadhyay, Ranjitsinh V Devkar

**Affiliations:** 1 BRD School of Biosciences, Sardar Patel Maidan, Vadtal Road, Satellite Campus, Post Box No. 39, Sardar Patel University,Vallabh Vidyanagar 388 120, Anand, Gujarat, India; 2 Division of Phytotherapeutics and Metabolic Endocrinology, Department of Zoology, Faculty of Science, The Maharaja Sayajirao University of Baroda, Vadodara-390002, Gujarat, India

**Keywords:** phycoerythrin, Phormidium sp. A27DM, Halomicronema sp. A32DM, A549 cell, apoptosis

## Abstract

Phycobilisomes present in cyanobacteria are photosynthetic macromolecular protein complexes that are categorized into three types - phycoerythrins (high energy), phycocyanin (intermediate energy) and allophycocyanin (low energy). Structurally, they consist of α and β protein subunits and open chain tetrapyrrole prosthetic group (bilin chromophore), known for its antioxidant properties and therapeutic potential against a variety of physiological ailments. Phycoerythrins (C-PE) were purified from cyanobacterial strains *Phormidium* sp. A27DM and *Halomicronema* sp. A32DM and their respective apoptotic potentials were assessed on A549 human lung carcinoma cells. Both strains of cyanobacteria were cultured and the C-PE from each strain was extracted, quantified and characterized. C-PE accounted for a dose dependent decrement in cell viability, mitochondrial membrane potential and an increment in lactate dehydrogenase release. Higher doses of C-PE (of both strains) accounted for loss of cell viability and nuclear pycnosis. These findings were further substantiated with flow cytometry that revealed a cell arrest at G_0_/G_1_ phase and a high percentage of cells undergoing apoptosis following C-PE treatment. These results confirm the efficacy of C-PE from *Phormidium* sp. or *Halomicronema* sp. in triggering apoptotic cell death. This study is the first to report on apoptotic property of C-PE against A549 human lung carcinoma cells and warrants further studies to establish its anti-cancer potential.

## Background

**Figure 1:**
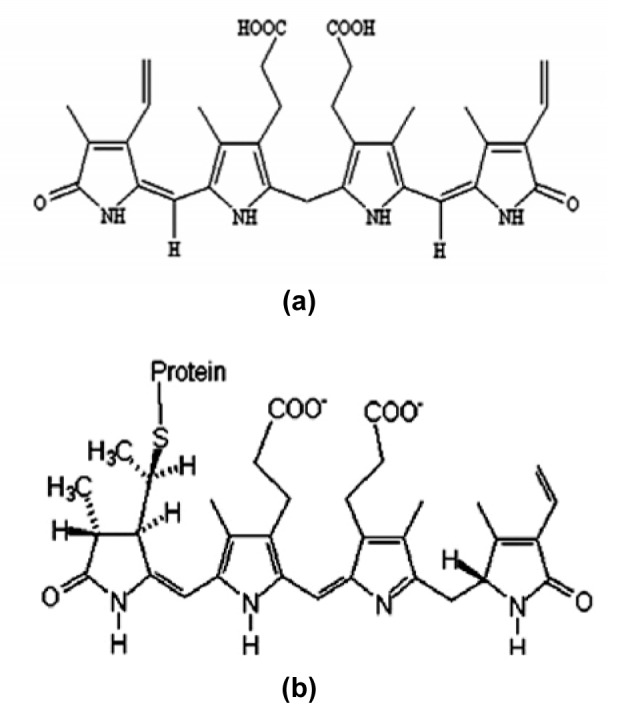
Chemical structure of phycoerythrin bilin chromophore (open-chain tetrapyrrol) (a) and bilirubin (b)

Cyanobacteria are organisms with an ability to carry out oxygenic photosynthesis, mechanistically identical with eukaryotic chloroplast-mediated photosynthesis. They are considered as the most primitive prokaryotes that evolved during the Precambrian period. In the last two decades, cyanobacteria has been at the epicenter of research due to their importance in human nutrition, agriculture, pharmaceutical industry and production of value added products of commercial importance. Phycobilisomes are macromolecular protein complexes that harvest light for the photosynthesis in cyanobacteria, cryptomonads and red algae (Berns and MacColl, 1989[5]). On the basis of bilin energy, phycobiliproteins or chromoproteins constituted within phycobilisomes, are categorized into three types - phycoerythrins (C-PE; high energy), phycocyanin (C-PC; intermediate energy) and allophycocyanin (C-APC; low energy) respectively. Structurally, they consist of α and β protein subunits and open chain tetrapyrrole prosthetic groups (bilin chromophore) (MacColl, 1998[19]). Structurally bilinchromophores in C-PE (Figure 1(Fig. 1)) share a similarity with bilirubin that is a degradative product of heme. Bilirubin has also been reported to exhibit potent antioxidant properties against reactive oxygen species (ROS), that are associated with a variety of patho-physiological processes including the ones seen during inflammation, neurodegenerative diseases, atherosclerosis, cancer or reperfusion injury (Kehrer, 1993[15]).

World Health Organization (WHO, 2009[30]), has established cancer to be one of the most common causes of death in developed countries and is also highly prominent in developing countries. Despite aggressive surgical and chemotherapeutic interventions, lung cancer remains the leading cause of cancer-related death worldwide with an estimated 90 % of the cases being tobacco related. Tobacco has been the epicenter of bronchogenic carcinoma accounting for 85-90 % of lung cancer deaths. Scientific surveys have also shown high levels of threats of lung cancer in non-smokers due to indirect exposure to tobacco smoke (Alberg et al., 2007[1]; Jemal et al., 2011[13]). The recent therapeutic advances and targeted therapies have resulted in poor survival statistics of 15.7 % on a five yearly basis. Hence, our understanding of tumerogenesis and related signaling pathways leading to onset and progression of cancer has led to a new ray of hope for the cancer patients.

Oxidative stress plays a key role in onset and progression of pathophysiological manifestation of many diseases including cancer. Intra-cellular oxidative stress is severally heightened under conditions of production of excessive ROS that cannot be mitigated by antioxidant defense system. Consumption of natural or synthetic antioxidants that can possibly scavenge free radicals has often been referred as an effective therapeutic option to alleviate ROS induced cellular damage. Studies suggest that phycobiliproteins from some algal species may inhibit growth of tumor cells (Huang et al., 2002[12]; Chen and Wong, 2008[7]) and C-PE, a natural antioxidant, is an efficient scavenger of oxygen free radicals (Belay, 2002[2]). C-PE from *Phormidium *sp. A27DM has been reported as potent hepto- and nephro-protective agent (Soni et al., 2009[24]), whereas, there are no reports on therapeutic potential of *Halomicronema* sp. A32DM. Preliminary studies on both *Phormidium *sp. A27DM and *Halomicronema* sp. A32DM in our laboratory reveled that they are both powerful antioxidant (data showcased herein). Hence, it was thought pertinent to access its apoptotic potential on A549 cells in culture through a series of carefully scripted *in vitro* protocols.

## Methods

### Chemicals

Sephadex powdered matrix (G-150; GE Healthcare UK Limited) with bead-diameter - 20-300 μm; fractionation range - 5 to 300 kDa, TPTZ 2, 4, 6-tripyridyl-s-triazine and ferric chloride (hexahydrate) from Sigma, USA; protein molecular mass standard (Bangalore Genei (Bangalore, India); sodium dodecyl sulphate (SDS), acrylamide and bisacrylamide (electrophoresis grade; Merck, Germany). Phosphate buffer saline(PBS)), The other chemicals and their sources of procurement were as follows: Dulbeco's Modified Eagle Medium (DMEM), trypsin phosphate versene glucose (TPVG) solution Trypsin and methylthiazolyldiphenyltetrazolium bromide (MTT) (Hi Media Laboratories Pvt. Ltd. Mumbai, India), Fetal bovine serum (FBS; Biosera, East Sussex, UK) and dimethyl sulfoxide (DMSO; Sisco Research Laboratories Pvt. Ltd. Mumbai, India).

### Cyanobacteria: isolation and growth conditions

The enrichment, isolation and purification of the cultures were carried out according to Shah et al. (2001[21]). The cyanobacterial samples collected from coasts of Devbhumi Dwarka, India and Daman Ganga estuary, Daman, India. Samples were further cultivated in ASN III medium (Waterbury and Stanier, 1981[29]) with 12:12 light:dark (L:D) cycles at 27 ° ± 2 °C and illumination of 36 W white fluorescent lamps at a flux density of 130 μmol photons.m^-2^.s ^-1^. Identification by 16S-rRNA gene sequencing showed that the isolated strains were *Phormidium* sp. A27DM (accession number - HM446281) and *Halomicronema* sp. A32DM (accession number - HM446282), referred in present study as *Phormidium* sp. and *Halomicronema* sp. respectively.

### Extraction and purification of phycoerythrin

#### Extraction

*Phormidium* sp. and *Halomicronema* sp. cultures were harvested within 30 days of inoculation (Soni et al., 2006[23]). Cyanobacterial cell mass were washed with 1 MTrisCl buffer (pH 8.1) with 3 mM sodium azide. Cell mass was resuspended in buffer (1:5; cells : buffer) and subjected to repeat freeze-thaw cycles (-30 °C and 4 °C). This caused release of phycobiliprotein which was collected and cell debris was removed by centrifugation at 17,000 x g (Kubota 6500, Bunkyo-Ku, Tokyo, Japan) for 40 min and the supernatant. The resultant product was termed as crude extract.

#### Purification

Steps of purification were carried out (Parmar et al., 2011[20]) for both *Phormidium *sp. and *Halomicronema* sp. All proceeding centrifugations were carried out at 15,000 x g for 40 min at 4 °C.

#### Ammonium sulphate precipitation

The crude extract was subjected to two step ammonium sulphate precipitation (20 % and 70 %) followed by centrifugation at 15,000 x g for 40 min at 4 °C. The pellet was resuspended in 10 mMTrisCl buffer (pH 8.1) and the resultant extract thus obtained were termed as 70 % ammonium sulphate cut (ASC) that was further subjected to dialysis for 24 hours against 100 times volume of MilliQ water containing 3 mM sodium azide.

#### Gel permeation chromatography

Dialysed 70 % ASC were applied on a Sephadex G-150 column (45cm x 1.5cm, bed height 35 cm) pre-equilibrated and eluted with 10 m*M* Tris Cl buffer (pH 8.1). The flow rate was maintained at 60 ml h^-1^ using peristaltic pump (Model P1; Pharmacia, Uppsala, Sweden). Bright pink colour elutes were collected as 1 ml fractions.

### Biochemical estimations and characterization of protein

The protein contents were determined by the method of Lowry et al. (1951[18]) with BSA as the standard. C-PE from both *Phormidium* sp. and *Halomicronema* sp. at all the three steps - crude, dialysed 70 % ASC and GPC elutes were characterized characterized by UV-Visible Spectrophotometer (Analytik Jena AG Specord^® ^210, Germany). The amount of C-PE, C-PC, and C-APC was calculated according to equations (Bennet and Bogorad, 1973[3]). The Ferric reducing ability of plasma (FRAP) assay was performed (Benzie and Strain, 1996[4]). A fresh cocktail solution was prepared by mixing solutions Solution A (300 µM acetate buffer - pH 3.6), Solution B (10 mM TPTZ 2, 4, 6-tripyridyl-s-triazine prepared in 40 Mm hydrochloric acid) and Solution C (20 mM ferric chloride hexahydrate) in the ratio of 10:1:1. Later, sample (30 µl) and distilled water (90 µl) was added to the resultant cocktail solution (900 µl). This mixture was incubated at 37 °C for 4 min and absorbance was read at 593 nm on UV-VIS spectrophotometer (Perkin-Elmer inc, USA).

Molecular masses of the purified C-PE from both *Phormidium* sp. and *Halomicronema* sp. were characterized. Non-denaturing and denaturing polyacrylamide gel electrophoresis (PAGE) was carried performed (Singh et al., 2009[22]) and visualized using silver staining (Garfin, 1990[10]). Marker proteins (range - 6 kDa to 43 kDa) were used for caliberation. There details are as follows: Aprotinin - 6 kDa, Lysozyme - 14.3 kDa, Soyabean Trypsin Inhibitor - 20.1 kDa, Carbonic Anhydrase - 29 kDa and Ovalbumin - 43 kDa. Bilinchromophore was detected by Zinc-acetate (Brekelman and Lagarias, 1986[6]), and the bilin fluorescence was observed under UV (Alpha Innotech Corp., San Leandro, CA, USA).

### Cell line and culture 

Human lung carcinoma (A549) cells were obtained from NCCS, Pune, India. All reagents were filtered through 0.22 μ filter (Laxbro Bio-medical Aids Pvt. Ltd, Pune) prior to their use for the experiment. Briefly, cells were seeded (1x 10^5^ cells/ T25 flask) and cultured in DMEM (with 10 % FBS and 1 % antibiotic-antimycotic solution) at 37 °C in CO_2_ incubator (Thermo scientific, forma series II 3110, USA). Cells were trypsinized (using TPVG solution) and sub-cultured every third day and maintained for a period of 24 h in absence or presence of C-PE from the two sources mentioned herein. MTT (cell density of 5.0 x 10^3^ cells/well in 96 well plate) and LDH assays (1 x 10^5^ cells/ well in 6 well plate), mitochondrial membrane potential assay, DCFDA and AO-EB staining, cell cycle analysis and Annexin V-PI staining assays were performed by standardized protocols.

### Effect of C-phycoerythrin on A549

#### Cell viability (MTT) assay 

A549 cells (7x10^3^ cells/ well) were maintained in 96-well culture plates (Tarson India Pvt. Ltd.) for 24 h with or without C-PE from *Phormidium* sp. or *Halomicronema* sp. (10, 25, 50, 100, 200 μg/ml). Later, 10 μl of 3-(4, 5-dimethylthiazol- 2-yl)-2, 5-diphenyl-tetrazolium bromide (MTT, 5 mg/ml) was added to the wells and plates were incubated at 37 °C for 4 h. Subsequently, the culture media were discarded and wells were washed with PBS. The resultant formazan formed was dissolved in 150 μl of DMSO and absorbance was read at 540 nm in ELX800 Universal Microplate Reader (USA). 

#### LDH release assay

Cellular integrity of A549 cells was measured with LDH release assay wherein; cells were maintained in 96 well plates with or without C-PE from* Phormidium* sp. or *Halomicronema* sp. for 24 h as mentioned above. Later, supernatant from each well was collected and LDH was assayed with commercially available kit (Reckon diagnostics Ltd., Mumbai, India) on Merck microlab L 300 semi-auto-analyzer (India). Based upon the readings obtained, percentage cytotoxicity was calculated.

#### Intracellular oxidative stress

Intracellular oxidative stress due to ROS generation in A549 cells was studied using DCFDA staining. Cells were treated with C-PE from *Phormidium* sp. or *Halomicronema* sp. for 18 h as per the method mentioned above. Later, the cells were incubated with 7.5 μM 2,7-dichlorodihydrofluoroscein diacetate (CM-H_2_-DCFDA) at 37 °C for 30 min and cells were photographed in Leica DMRB fluorescent microscope (Germany) using Canon PowerShot camera.

#### Mitochondrial membrane potential

Possible alterations in mitochondrial membrane potential were assessed using Rhodamine 123 dye (RHO 123). A549 cells were incubated for 24 h with or without C-PE from *Phormidium* sp. or *Halomicronema* sp. as mentioned above. Later, cells were incubated with 1 μM RHO 123 for 10 min and the fluorescence was determined at 485 and 530 nm, respectively using spectroflurometer (Jasco FP-6300, Japan) and expressed as fluorescence intensity units (FIU).

### Assessment of apoptosis and cell cycle analysis

A549 cells (5 x 10^4^ cells/well) were seeded into 6-well plate and allowed to attain 80 % confluence. The cells were cultured in presence or in absence of various concentrations of C-PE from *Phormidium* sp. or *Halomicronema* sp. at 37 °C for 24 h. Possible alterations in nuclear morphology was done using DAPI staining. Single-cell suspensions of treated cells were washed with PBS and fixed with 70 % ethanol for 20 min at room temperature. Cells were re-washed with PBS and stained with DAPI (0.6 *μ*g/mL in PBS) for 5 min. Nuclear morphology (condensed/fragmented nuclei) was examined under Leica DMRB fluorescence microscope (Germany).

Apoptotic cells were studied using AO/EB staining. A549 cells (1 x 10^5 ^cells/ well) were maintained in 6 well plates for 24 h with or without C-PE from *Phormidium* sp. or *Halomicronema* sp. as described earlier. Cells were collected using TPVG solution and 9 μl of cell suspension (0.5 x 10^6^ cells/ml) was treated with 1 mg/ml AO and 1 mg/ml EB in PBS on a clean microscope slide. Observations were recorded and evidences were photographed under Leica DMRBfluorescence microscope (Germany). 

Cells (1 x 10^6^ cells/well) were cultured with or without PE as mentioned earlier for 24 h. Cell cycle analysis was done for cells as per standard procedure. Briefly, 1 x 10^5^ cells were fixed in 4.5 ml of 70 % (v/v) cold ethanol for 30 min. This mixture was then centrifuged at 400 g for 5 min. Cells were re-washed with 5 ml of PBS and re-suspended in 0.5 ml of PBS and 0.5 ml of DNA extraction buffer. A mixture of 192 ml of 0.2 M Na2HPO4 with 8 ml of 0.1 % Triton X-100 v/v was added so as to achieve a pH of 7.8. Cells were briefly incubated and then centrifuged (400 g for 5 min) re-suspended in 1 ml of DNA staining solution (200 mg of PI in 10 ml of PBS + 2 mg of DNase free RNase). After 30 min of incubation in the dark, cells were subjected to flow cytometeric analysis (BD FACS Aria III, USA) using FlowJo (Oregon, USA).

Annexin-V/Propidium iodide double staining assay was used to quantify apoptosis, according to the manufacturer's protocol (Alexa Fluor 488 annexin V/Dead Cell Apoptosis Kit, Invitrogen). After incubation, cells were harvested using TPVG solution and washed with ice-cold PBS and suspended in 100 μl of 1x binding buffer (10 mM HEPES, 140 mM NaCl, and 2.5 mM CaCl2, pH 7.4). To this mixture, 5 μl of annexin V- Alexa Fluor 488 conjugate and 1 μl of propidium iodide solution were added to each cell suspension and incubated for 15 min at room temperature in the dark. Samples were analyzed on flow cytometer (BD FACSAria III, USA) using FlowJo (Oregon, USA). Double staining of cells with Alexa Fluor 488 Annexin-V and PI enables the discrimination of live cells (Alexa Fluor 488^-^PI^-^), early apoptotic (Alexa Fluor 488^+^PI^-^), late apoptotic (Alexa Fluor 488^+^PI^+^) or necrotic cells (Alexa Fluor 488^-^PI^+^).

### Statistical analysis 

Statistical significance using one way analysis of variance (ANOVA) was employed to analyze the data followed by Bonferroni's multiple comparison test. Results were expressed as mean ± SEM using Graph Pad Prism version 3.0 for Windows, Graph Pad Software, San Diego, California, USA.

## Results

### Growth of cyanobacteria 

Growth of *Phormidium* sp. and *Halomicronema* sp. were monitored at every 48 h of incubation. 50 mg cell mass was harvested by centrifugation and its phycoerythin and chlorophyll *a* contents were analyzed. No increment could be recorded for the first 4 days of incubation, but subsequent samples recorded growth linked concomitant increase in phycoerythrin content that was maximum on 30^th^ day of incubation.

### Extraction and purification of phycoerythrin

**Figure 2:**
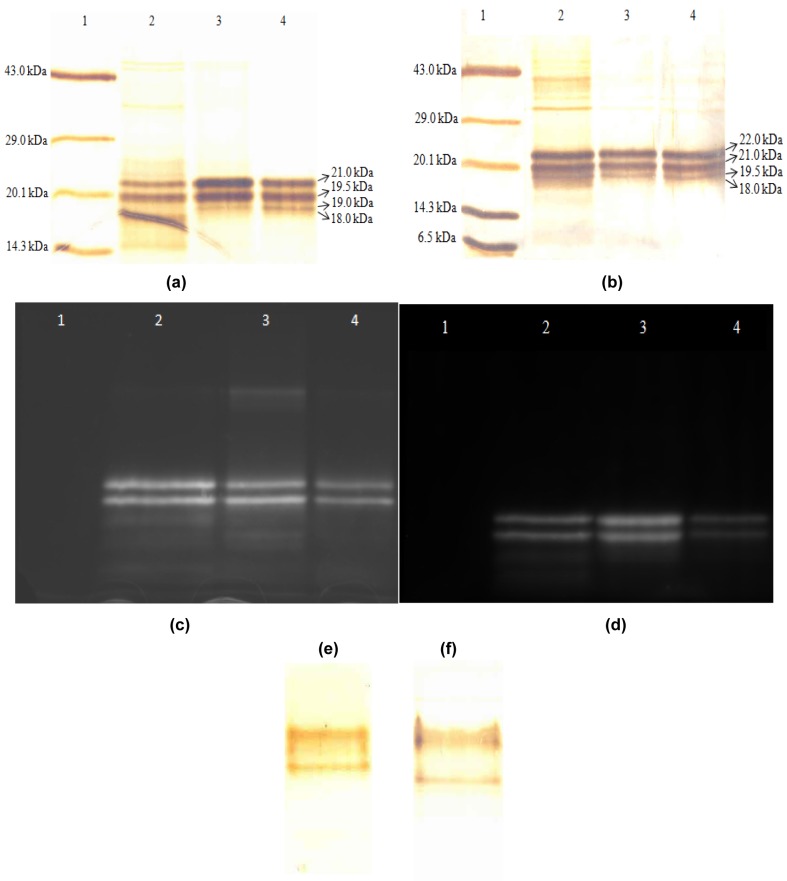
Silver stained 15 % resolving gel of SDS-PAGE of protein from Phormidium sp. A27DM (a) and Halomicronema sp. A32DM (b). Detection of biliproteins from Phormidium sp. A27DM (c) and Halomicronema sp. A32DM (d) by zinc-assisted fluorescence enhancement method at each step of purification as observed under UV light. For (a), (b), (c) and (d) Lanes: 1. Protein molecular mass marker; 2. Crude extract; 3. 70 % ASC; 4. Pure PE eluate from gel permeation chromatography. There were no biliproteins present in molecular mass marker and so no fluorescence observed indicated the specificity of the method for biliprotein detection. The protein loaded was 5 µg in each lane. Silver stained 12 % Native gel electrophoresis of purified protein from Phormidium sp. A27DM (e) and Halomicronema sp. A32DM (f).

Extraction of C-PE from *Phormidium* sp. and *Halomicronema* sp. could be achieved via four cycles of freezing and thawing (-30 °C and 4 °C respectively). The crude extract (stage I) obtained herein was further centrifuged (15,000 x g for 30 min) and the resultant supernatant showed highest absorption peak at 562 nm that suggested a high content of C-PE as compared to C-PC or C-APC. Further confirmation for the same was obtained by silver stained gel electrophoresis (Figures 2a and b(Fig. 2)). At this step (stage II), purity ratio of C-PE was 2.77 and 1.46 for *Phormidium* sp. and *Halomicronema* sp. respectively. Fractionation of dialyzed C-PE with Sephadex G-150 further increased the purification levels to 4.26 and 3.73 respectively (stage III). The final C-PE contents in precipitates of *Phormidium* sp. and *Halomicronema* were 83.2 % and 72.1 % respectively (Table 1(Tab. 1) and 2(Tab. 2)).

### Characterization

At each step, C-PE purification was monitored with UV-Visible spectroscopy and gel electrophoresis.

#### Spectral analyses

**Figure 3:**
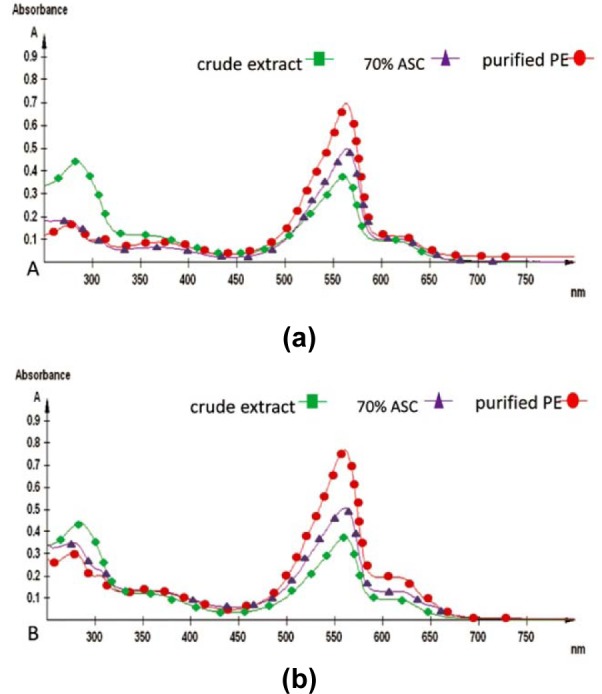
UV-Visible absorption overlay spectra of C-phycoerythrin from Phormidium sp. A27DM (a) and Halomicronema sp. A32DM (b) at each step of purification.

UV-Visible spectroscopy showed 562 nm as the absorbance maxima (λ_max_) of C-PE (Figure 3(Fig. 3)). The purity of C-PE from both *Phormidium* sp. and *Halomicronema* sp. at each step of purification was recorded in terms of purity ratio (*A*_562_/*A*_280_) (Table 1(Tab. 1) and 2(Tab. 2)). The purity ratio of C-PE to total protein (*A*_562_/*A*_280_), C-PE to C-PC (*A*_562_/*A*_620_), and C-PE to C-APC (*A*_562_/*A*_650_) was 4.26, 6.22, and 13.58, respectively for *Phormidium* sp. (Table 1(Tab. 1)) and 3.73, 4.03, and 9.44, respectively for *Halomicronema* sp. (Table 2(Tab. 2)). The increase in the purity ratio at each step of purification is shown in overlay spectra for *Phormidium* sp. (Figure 3a(Fig. 3)) and *Halomicronema* sp. (Figure 3b(Fig. 3)) recorded at 562 nm.

#### Gel electrophoresis

Non-denaturing gel electrophoresis of purified C-PE showed two bands each for *Phormidium* sp. (Figure 2e(Fig. 2)) and *Halomicronema* sp. (Figure 2f(Fig. 2)), probably corresponding to PE-I and PE-II. These results also suggested homogeneity of purified C-PE. Denaturing gel electrophoresis of C-PE showed presence of four bands each with molecular mass of 21, 19.5, 19, and 18 kDa for *Phormidium* sp. (Figure 2a(Fig. 2)) and 22, 21, 19.5, and 18 kDa for *Halomicronema* sp. (Figure 2b(Fig. 2)). 

Bilin of the protein fluoresce orange in the presence of zinc ions under the UV radiation. The presence of bilin chromophore group attached to the PE subunit was confirmed by zinc-acetate staining that showed characteristic orange fluorescence under UV light. no fluorescence was observed in molecular mass standard due to the absence of bilin-linked peptides. These results also confirmed attachment of bilin chromophore groups to the subunits of C-PE for both *Phormidium* sp. (Figure 2c(Fig. 2)) and *Halomicronema* sp. (Figure 2d(Fig. 2)).

#### Determination of antioxidant capacity

FRAP assay show the antioxidant capacity of purified C-PE from both *Phormidium* sp. and *Halomicronema* sp. was 50.60 µM AEAC and 47.45 µM AEAC respectively suggesting the potential use of phycoerythrin as an antioxidant protein in therapeutics.

### Effect of C-phycoerythrin on A549

#### Cytotoxicity assay and LDH release

**Figure 4:**
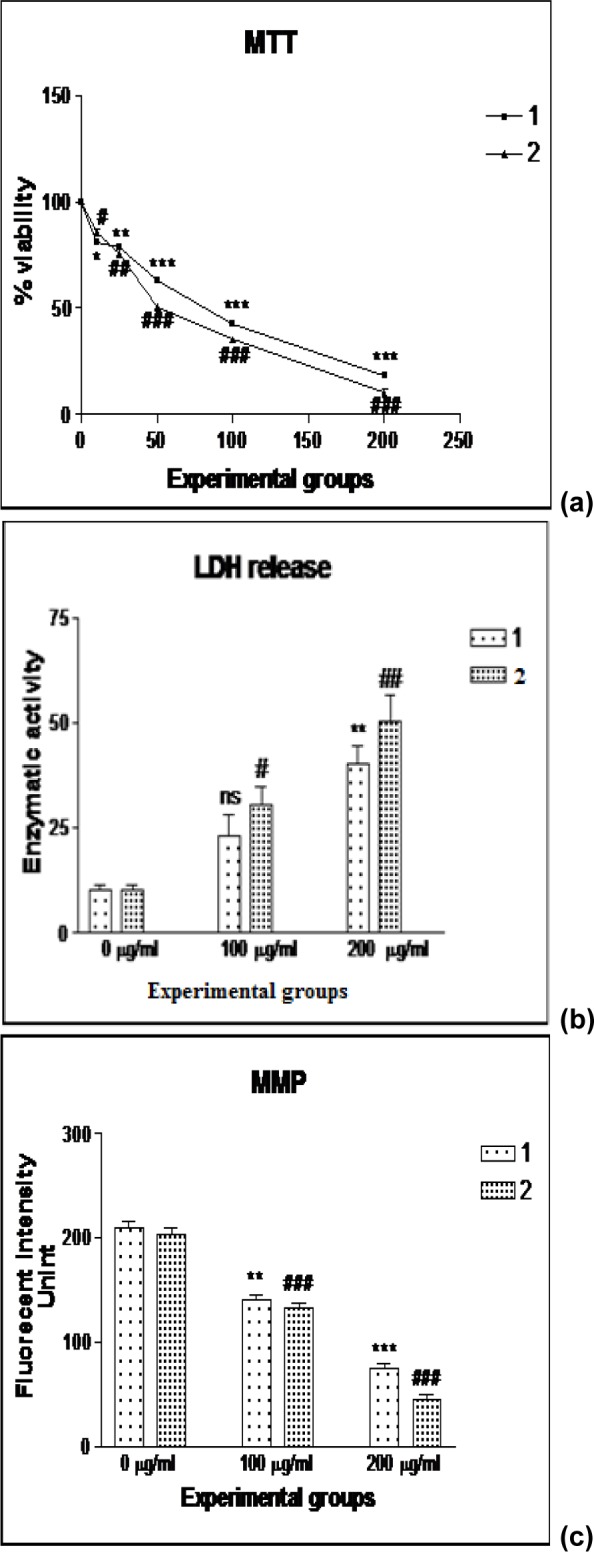
Effect of C-PE from Phormidium sp. A27DM (1) and Halomicronema sp. A32DM (2) on cell proliferation of A549 cell lines upon treatment with various concentration for 24 h (a), on LDH release (b), and mitochondrial membrane potential (c), as compared with untreated cells. The significant difference between the C-phycoerythrin treatment from both strains and the control is indicated at *P < 0.05; **P < 0.01; ***P < 0.001

Cytotoxic assay (Figure 4a(Fig. 4)) of C-PE from *Phormidium* sp. or *Halomicronema* sp. against A549 cells showed a dose dependent inhibitory effect with the higher doses (100 and 200 µg/ml) being the most effective. Also, there was a significant dose dependent increase in LDL release (Figure 4b(Fig. 4)) following *Phormidium* sp. or *Halomicronema* sp. treatments (200 µg/ml).

#### Mitochondrial membrane potential

The MMP was assayed by fluorimetric method for untreated and C-PE from *Phormidium* sp. or *Halomicronema* sp. treated A549 cells (Figure 4c(Fig. 4)). The same showed a significant dose dependent decrement in mitochondrial membrane potential.

### Assessment of apoptosis and cell cycle analysis

**Figure 5:**
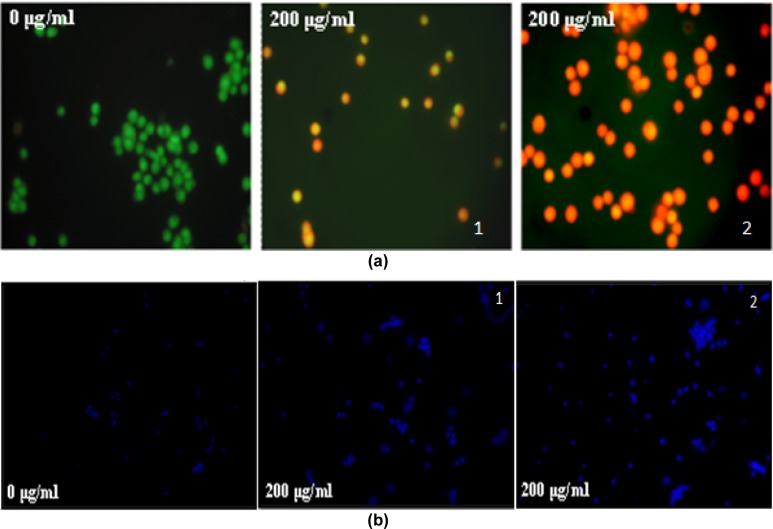
Representative fluorescence images of C-PE from Phormidium sp. A27DM (1) and Halomicronema sp. A32DM (2) induced DNA condensation/ fragmentation in A549 cells as detected by AO/EB staining (a) and DAPI (b) staining (40X). Cells were cultured with or without 200 µg/ml purified C-PE from both strains for 24 h.

Visual evidences of possible loss of cell viability were obtained using AO/EtBr staining. The untreated cells showed green fluorescence (viable cells) whereas, C-PE from *Phormidium* sp. or *Halomicronema* sp. treatment accounted for more number of yellowish to red coloured cells (dead cells). Higher dose of C-PE from* Phormidium* sp. or *Halomicronema* sp. (200 µg/ml) showed more number of reddish or orange cells indicating late apoptotic phase (Figure 5a(Fig. 5)).

**Figure 6:**
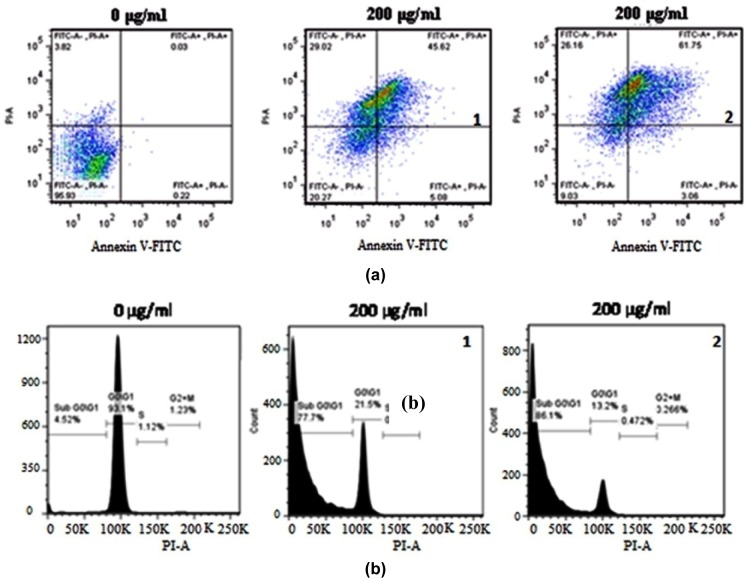
Cells treated with 200 µg/ml purified C-PE from Phormidium sp. A27DM (1) and Halomicronema sp. A32DM (2) for 24 h were harvested and stained with propidium iodide and the DNA content was quantified by FACS (a), also stained with Annexin V-FITC/PI and then analysed by flow cytometry (b). 0 µg/ml - Untreated A549 cells.

Nuclear condensation following C-PE from *Phormidium* sp. or *Halomicronema* sp. treatment was studied using DAPI staining. Higher dose of C-PE from *Phormidium* sp. or *Halomicronema* sp. (200 µg/ml) showed prominent nuclear condensation (Figure 5b(Fig. 5)). Cell cycle analysis showed higher percentage of cells following C-PE from *Phormidium* sp. (77.7 %) or *Halomicronema* sp. (86.1 %) treatment to A549 cells (Figure 6a(Fig. 6)). Flow cytometric analysis using Annexin V/PI double staining of control and treated cells showed that C-PE from *Phormidium* sp. (45.62 %) or *Halomicronema* sp. (61.75 %) accounted for higher percentage of late apoptotic cells (Figure 6b(Fig. 6)).

## Discussion

Isolation, purification and assessment of efficacy of natural substances in inhibiting carcinogenesis are currently in the focus of research in cancer biology (Gorczyca et al., 1993[11], Thounaojam et al., 2011[25]; Valodkar et al., 2011[26], 2012[27]; Vyas et al., 2013[28]). Polysaccharides, peptides and phycobiliproteins extracted from algal sources have been put to scrutiny to assess their antitumor potential and to decipher their mode of action (Liu and Rein, 2010[17]; Zhang et al., 2011[31]). In this regard, induction of cell apoptosis by these bioactive substances have been assessed in detail as the same is thought to be a key mechanism in preventing tumorogenesis. In an effort to gain an insight into the effect of C-PE from *Phormidium* sp. or *Halomicronema* sp. have been put to scrutiny via a series of experimental protocols using A549 lung carcinoma cells to establish their anti-cancer potential. To begin with, C-PE from both *Phormidium* sp. or *Halomicronema* sp. was extracted efficiently by freezing and thawing. High contents of C-PE obtained at the end of extraction and purification process is attributable to the modifications of the protocol mentioned herein and hence, can be considered as an efficient method for production of C-PE.

Anti-cancer compounds are instrumental in terminating cell proliferation in multiple ways such as tubulin binding (G2/M arrest), actin binding, and inhibition of CDKs and trigger apoptosis (Thounaojam et al., 2011[25]). Cytotoxicity assay revealed that both C-PE from *Phormidium* sp. or *Halomicronema* sp. were able to manifest cell death as evidenced by the data obtained in MTT assay. A higher dose of 200 µg/ml was most potent as it accounted for maximum cell growth inhibition (> 80 %). Biochemical estimation of LDH is a reliable and popular marker that indicates cell damage (Thounaojam et al., 2011[25]). This is because any damage to the plasma membrane of an animal cell causes leaching out of LDH from cytoplasm into the media and the same is measured as an indication of the extant of cell damage (Devkar et al., 2012[8]). LDH levels were significantly elevated following C-PE treatments that further confirmed cellular stress and damage. High levels of LDH are also in agreement with the results observed in MTT assay. 

Also, C-PE from Phormidium sp. or Halomicronema sp. treatment accounted for alteration of transmembrane potential of mitochondria amounting to an overall decrement in MMP. Assessment of mitochondrial function using MTT assay is an established protocol for assessing cytotoxicity where as, LDH release indicates compromised cellular integrity (Devkar et al., 2012[8]). Hence, in present study the result clearly suggests occurrence of cell death following C-PE from Phormidium sp. or Halomicronema sp. treatment due to mitochondrial dysfunction, damage of the cell membrane and possibly due to trigger of apoptotic cascade. Trigger of apoptosis following treatment with bioactive substances is well documented in cancer cell lines. This is because altered membrane permeability of mitochondria and leaching of cytochrome C into the cytoplasm from the transitional pores has been proved to trigger apoptosis in cancer cells (Jiang and Wang, 2004[14]). In our study, we have observed higher number of EtBr positive (orange/red) cells following C-PE from Phormidium sp. or Halomicronema sp. treatment. These observations suggest loss of cell viability and further corroborate the findings of MTT assay. During apoptosis, nuclear shrinkage, chromatin condensation, nuclear fragmentation and formation of apoptotic bodies are commonly observed features (Elmore, 2007[9]), and the same could be observed in DAPI stained C-PE from Phormidium sp. or Halomicronema sp. treated cells. Flow cytometry of untreated and C-PE from Phormidium sp. or Halomicronema sp. treated cells provided further evidence in form of a cell cycle arrest in G0/G1 phase. Also, Annexin V FITC/ PI assay showed significantly higher percentage of late apoptotic cells following C-PE treatment. Other research group has reported that cytochrome C in cytosol activates caspases that cleaves cellular substrates and accounts for apopotic cell death (Li et al., 2006[16]) and the same is assumed in our findings. These results confirmed the efficacy of C-PE from Phormidium sp. or Halomicronema sp. in triggering apoptotic cell death.

## Conclusion

This study is the first to characterize C-PE from *Phormidium* sp. or *Halomicronema* sp. and validate their apoptotic property against A549 human lung carcinoma cells. Encouraging results obtained herein are attributable to C-PE induced cell death and warrants further studies to establish its anti-cancer potential.

### Conflict of interest

The authors declare no conflict of interest.

### Abbreviations

C-PE, Phycoerythrins, A27DM, *Phormidium* sp. accession number, A32DM, *Halomicronema* sp. accession number, A549, human lung carcinoma cells, C-PC, phycocyanin, C-APC, allophycocyanin, ROS, Reactive oxygen species, TPTZ 2, 4, 6-tripyridyl-s-triazine, PBS , Phosphate buffer saline, DMEM, Dulbeco's Modified Eagle Medium, TPVG, trypsin phosphate versene glucose, MTT, ethylthiazolyldiphenyl- tetrazolium bromide, FBS, Fetal bovine serum, DMSO, dimethyl sulfoxide, ASC, ammonium sulphate cut, GPC, gel permeation chromatography, FRAP, Ferric reducing ability of plasma, LDH, lactate dehydrogenase, **DCFDA**, 2',7'-dichlorofluorescein diacetate, **AO/EB**, Acridine orange/ethidium bromide, PI, propidium iodide, CM-H2-DCFDA, 2,7-dichlorodihydrofluoroscein diacetate, RHO 123, Rhodamine 123, DAPI - 4',6' diamino-2-phenylindole, FACS, Fluorescent activated cell sorting, Na2HPO4, disodium hydrogen phosphate, CaCl2, calcium chloride, HEPES, (4-(2-hydroxyethyl)-1-piperazineethanesulfonic acid, ANOVA, analysis of variance, EtBr, ethidium bromide.

### Acknowledgments

RVD acknowledges UGC for financial support in form of Major research project No.F.41-89/2012(SR) and MSU-DBT-ILSPARE for infrastructural and technical support. Help rendered by Avani Kaushal in performing wet lab experiments is duly acknowledged.

## Figures and Tables

**Table 1:**
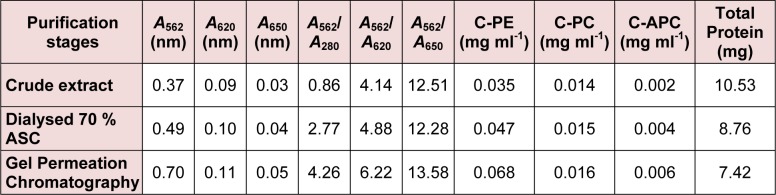
Determination of spectophotometric purity and concentration of phycobiliproteins from *Phormidium* sp. A27DM

**Table 2:**
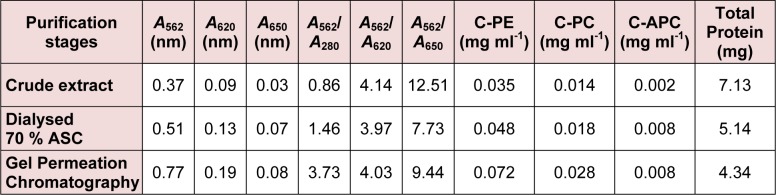
Determination of spectophotometric purity and concentration of phycobiliproteins from *Halomicronema* sp. A32DM
